# Bioinformatic assessment of the potential amyloidogenicity of the human and evolutionarily more ancient proteomes

**DOI:** 10.1042/BCJ20250336

**Published:** 2026-07-02

**Authors:** Ivayla Roberts, Xiaomian Tan, J. Bernadette Moore, Etheresia Pretorius, Douglas B. Kell

**Affiliations:** 1 Department of Biochemistry, Cell and Systems Biology, Institute of Systems, Molecular and Integrative Biology, University of Liverpool, Crown St, Liverpool L69 7ZB, U.K.; 2 Department of Physiological Sciences, Faculty of Science, Stellenbosch University, Stellenbosch Private Bag X1 Matieland, 7602, South Africa; 3 The Novo Nordisk Foundation Centre for Biosustainability, Building 220, Søltofts Plads 200, Technical University of Denmark, 2800 Kongens Lyngby, Denmark

**Keywords:** AmyloGram, Amyloidogenesis, evolutionary biology, protein aggregation

## Abstract

AmyloGram is a computer program that uses n-gram encoding and a random forest classifier to produce a numerical score between 0 and 1 for the predicted amyloidogenicity of a given protein sequence. In a variety of recent studies, we have used AmyloGram to obtain an overall amyloidogenicity score for members of the human proteome. Of 83,567 full-length canonical human polypeptides, 79.2% had a score exceeding 0.7 (the median was 0.813), consistent with the view that most natural protein sequences contain elements that are in fact potentially amyloidogenic. Here, we first asked whether this operational threshold is supported by orthogonal predictors and curated amyloid proteins, and then whether similarly high scores are also observed in evolutionarily ancient proteomes. For the human proteome, PASTA2 values correlated positively with AmyloGram scores (*r*^2^ = 0.374 for minimum free energy and 0.321 for average free energy), and proteins with AmyloGram scores ≥0.7 were significantly enriched for strongly negative PASTA2 values (for average free energy <−10 PEU: 4724/66190 versus 75/17,377; χ^2^ *P* = 2.4 × 10^−250^). In AmyPro, 117 curated amyloid proteins had substantially higher median AmyloGram scores than did the ~8 curated non-amyloids, although the imbalance of this dataset demands caution. AMYPred-FRL showed only a weak and partly discordant relationship with AmyloGram in the archaeal test proteome examined. We then computed AmyloGram score distributions for the proteomes of 130 other organisms, including archaea, Gram-negative bacteria, Gram-positive bacteria and viruses, representing 475,999 proteins in total. The corresponding organism-level median AmyloGram scores by domain were 0.822, 0.853, 0.851, and 0.825, respectively, while the overall median across all 130 organism-level medians was 0.828. By contrast, 1000 random 100-mer sequences generated with equal amino-acid probabilities had a median AmyloGram score of 0.740. Weighting according to known residue distributions did not change this. However, a weak dependence of AmyloGram score on sequence length within an organism was observed, and the median length of human proteins (∼375 residues) gave a median AmyloGram score of 0.837. Taken together, these findings are consistent with, though they cannot prove, an early evolutionary origin of widespread amyloidogenic potential in natural proteins, and most likely reflect the biophysical properties of the amino acid residues that tend to induce amyloidogenesis.

## Introduction

A variety of chronic, inflammatory diseases are recognised as being accompanied by the deposition of particular proteins in an alternative, amyloid form, that consists of insoluble fibrils. This amyloid form has a defining structural motif, the cross-β motif [1–8], which consists of β-sheets aligned perpendicular to the fibril axis and which makes these forms rather resistant to proteolysis. Such diseases are known as amyloidoses (e.g [9–23]). This said, certain other amyloids are considered beneficial, in that they are known to be involved in specific signalling and other functional pathways [24–28].

The amyloids formed in many cases contain multiple proteins, due to the phenomenon of cross-seeding [29–37], in which the presence of a particularly amyloidogenic protein flips others into an amyloid form that can become incorporated into the growing fibrils.

We have discovered that in a number of cases [38–48] the polymerisation of fibrinogen during blood clotting can cause it too to fold into an amyloid form. As is well known, amyloid forms of proteins are typically rather resistant to agents that would normally catalyse their proteolysis (e.g [38,49–59]), and those thrombi formed in the presence of the SARS-CoV-2 spike protein [42–44] or certain peptides therefrom [60] are especially resistant to fibrinolysis [61].

The amyloidogenicity of a protein can be assessed from its sequence (see also [62,63]), and, as part of these studies, we have looked at the amyloidogenic potential of specific proteins [36,48,64], mostly using the computer program AmyloGram [65,66]. This program was chosen over the various others available (as listed in [35]) for three reasons that were rehearsed in [48]: (i) it demonstrated the highest predictive performance in its original validation [65]; (ii) it provides a single, interpretable score ranging from 0 to 1; and (iii) it is available (see the ‘Materials and methods’ section) both as a web application and as R code. Recently, we have used it extensively to assess the amyloidogenicity of proteins found in thrombi from ischaemic stroke [48] and in insoluble disease-associated aggregates more generally [67]. We calibrated the system using proteins known to be amyloidogenic (and annotated as such at UniProt), which indicated that essentially all have AmyloGram scores exceeding 0.7. The former paper also included an analysis of 83,567 full-length canonical human polypeptides in UniProt, finding that 79.2% of sequences gave an AmyloGram score above 0.7, confirming a widespread presence of amyloidogenic motifs in the majority of natural proteins (see also [63,68–82]). In short, most proteins seemingly can be induced to form amyloid fibrils, whether alone or by cross-seeding. Later, we shall investigate amyloidogenicity for a wide variety of proteomes.

Despite the reasons we have stated behind the utility of AmyloGram, a possible criticism is that we did not assess amyloidogenicity using other sequence-based programs (some are structure based, but since most proteins lack experimental 3D structures they are not considered here). These analyses left at least two open questions. The first was how comparable were the results from AmyloGram, which has the benefit of returning a single score between 0 and 1, with any other of the various amyloid-predicting programs. In the event, most of these focused on the identification of hot spots, and we felt that PASTA2.0 was the best candidate for comparison, not least as it uses an entirely different free energy basis for assessing its scores. Thus, the first purpose of the present work is to extend our analysis to other methods of amyloidogenic assessment. We also recognise that our analyses had focused entirely on the human proteome, and it was of interest to know how common the occurrence of amyloid was likely to be in other taxa. Thus, we also applied AmyloGram to a much wider range of proteomes from different taxa.

## Results

### Analysing AmyloGram thresholds

**Figure 1:**
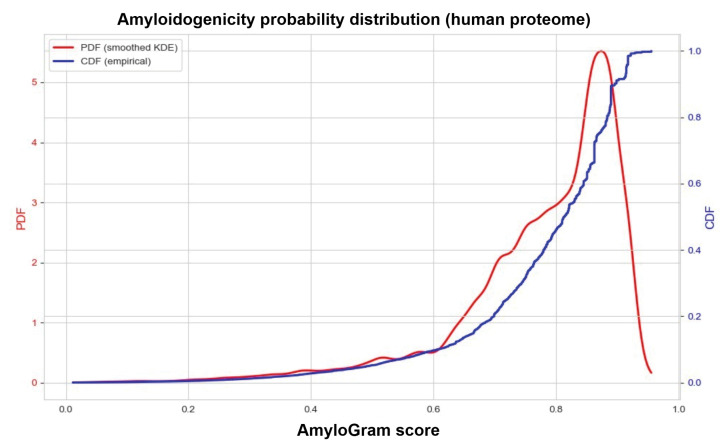
Probability (PDF) and cumulative (CDF) distribution functions for the AmyloGram scores of 83,657 full-length canonical human polypeptides in the human proteome [67] The Amylogram scores were rank-ordered using the data in the supplementary information of reference [67]. AmyloGram returns a score between 0 and 1, with higher values indicating greater predicted amyloidogenic propensity. The PDF was smoothed using Python’s Kernel Density Estimator in the seaborn (0.13.2) library. This figure is included to orient the reader to the previously observed score distribution in the human proteome and to show why the threshold of 0.7 was taken forward for the present benchmarking and evolutionary analyses.

Figure 1 first displays the data for the 83,567 full-length canonical human polypeptides studied previously [67] as probability and cumulative distribution functions, reprising the dot plot previously published [67] (with a median of 0.813, as may also be judged from Figure 4A of [67]) but here in a rather more convenient format. The distribution is strongly shifted towards higher scores, with a median of 0.813, and 79.2% of proteins scoring above the previously adopted operational threshold of 0.7.

### Comparing AmyloGram and PASTA 2.0 amyloidogenicity prediction

We next looked through the various amyloid prediction servers that exist to find those with straightforward outputs suitable for comparison with the simple numerical outputs of AmyloGram. Many would provide detailed comparison of the amyloidogenicity of various segments (as will AmyloGram itself) but these are very specific to the precise sequence of the individual proteins. Perhaps surprisingly, the only one that we could find to be really suitable for us was the PASTA 2.0 [83].

PASTA recognises that the biophysical basis of the tendency of polypeptides to aggregate is broadly similar to that of the tendency to form amyloid (see also [32,84–97]), and PASTA algorithms calculate a corresponding free energy function (the more negative values relating to an increased aggregation/amyloidogenic tendency). The PASTA 2.0 energy function evaluates the stability of putative cross-beta pairings between different sequence stretches [69,83]. Consequently, the free energy function from PASTA2 can provide a single value that might be compared with the AmyloGram score. The PASTA algorithm and server predicts aggregation in energy units where 1 PASTA Energy Unit (PEU) is stated to be ‘equivalent to 2 k_B_T at room temperature, that is 1.192 Kcal*/*mol’ [83].

**Figure 2:**
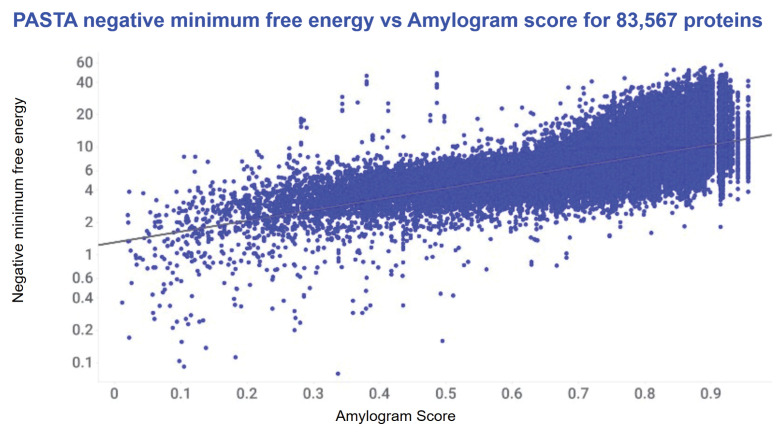
Relationship between AmyloGram scores and PASTA2 minimum free energies in the human proteome Scatter plot comparing AmyloGram scores with the negative of the minimum PASTA2 free energy for 83,567 human proteins and polypeptides. PASTA2 reports energies in PEU, with more negative raw values indicating greater predicted aggregation propensity; the sign is inverted here so that larger plotted values correspond visually to stronger predicted amyloid/aggregation tendency and are therefore easier to compare with AmyloGram scores. Each point represents one protein. The fitted linear regression is shown, with *r*^2^ = 0.374. This figure is intended as an orthogonal benchmarking comparison rather than as a ground-truth validation. It shows that proteins with higher AmyloGram scores tend, on average, also to have more negative minimum PASTA2 energies.

Figure 2 shows the minimum free energy plotted (without its negative sign) against the AmyloGram scores (taken from [48]) for the 83,567 human polypeptides and proteins, with a clear positive correlation, that has an *r*^2^ of 0.374.

When the proteins are stratified by the previously defined AmyloGram threshold of 0.7, 20,769/66,190 (31.4%) of those above threshold have minimum PASTA2 free energies lower than −10 PEU, compared with only 393/17,377 (2.26%) below threshold; for a threshold of −20 PEU the corresponding numbers are 4872/66,190 (7.36%) and 28/17,377 (0.16%). Both enrichments are highly significant (χ^2^ *P* < 10^−250^ and *P* = 9.4 × 10^−283^, respectively). These data support the view that proteins assigned high AmyloGram scores are, on average, also assigned markedly more aggregation-prone free energies by the orthogonal PASTA2 framework.

**Figure 3:**
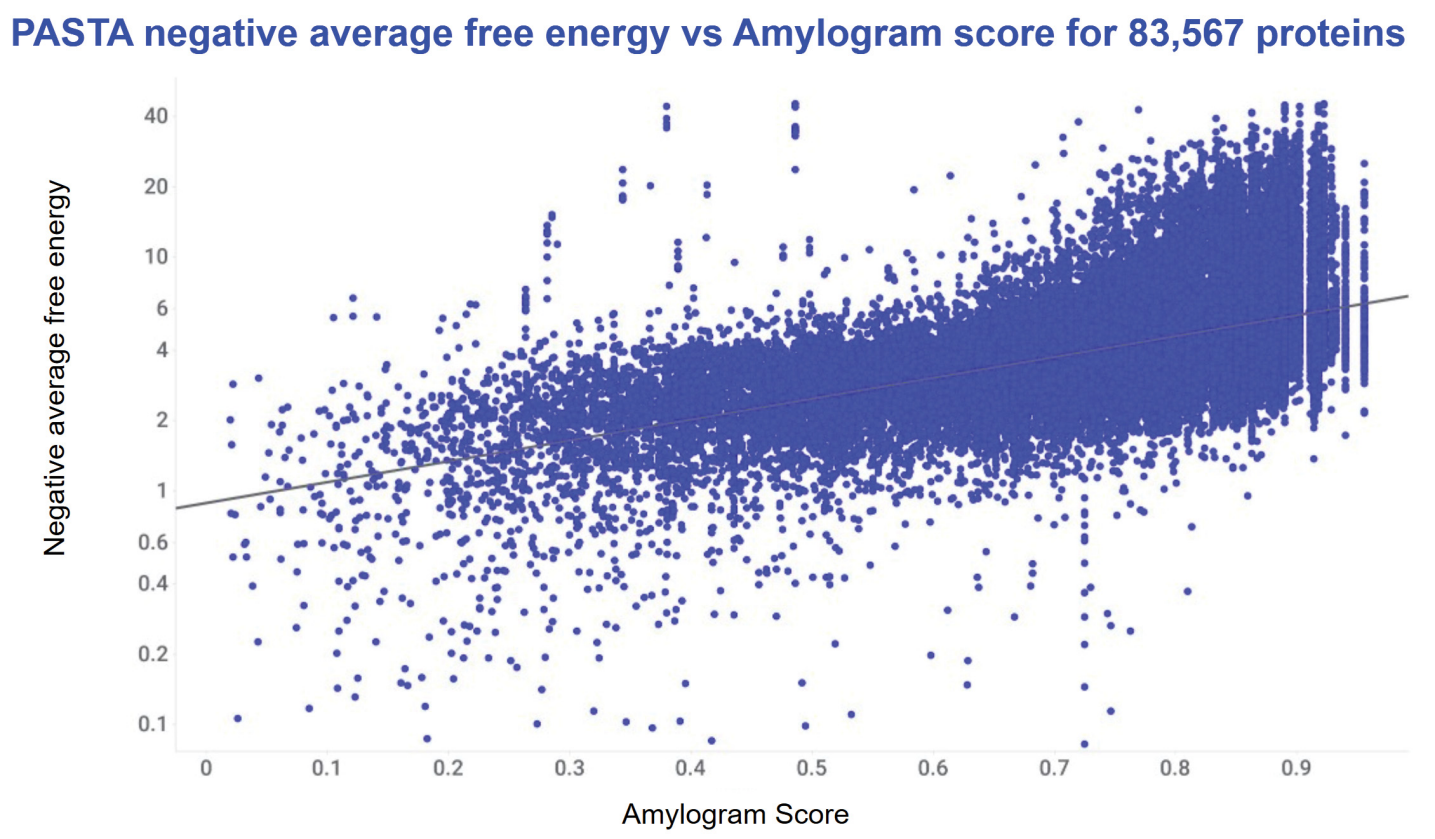
Relationship between AmyloGram scores and PASTA2 average free energies in the human proteome Scatter plot comparing AmyloGram cores with the negative of the average PASTA2 free energy for the same 83,567 human proteins and polypeptides analysed in Figure 2. As in Figure 2, the sign of the PASTA2 output is inverted for plotting so that higher values correspond to more negative raw PASTA2 energies. Each point represents one protein, and the fitted linear regression is shown (*r*^2^ = 0.321). The association is somewhat weaker than that seen for minimum free energy, but remains clearly positive overall. This figure therefore provides additional benchmarking support that proteins assigned higher AmyloGram scores are enriched for more aggregation-prone PASTA2 energy profiles, while also underscoring that the two methods are not interchangeable and capture overlapping rather than identical aspects of amyloidogenic potential.

Figure 3 shows data from the same comparison as that in Figure 2, but instead uses the (negative of) the average free energy for each protein in the PASTA data. The linear relationship remains positive, though weaker (*r*^2^ = 0.321). Of the 66,190 proteins with an AmyloGram score of at least 0.7, 4724 (7.14%) have an average free energy lower than −10 PEU and 728 (1.10%) lower than −20 PEU, whereas for the 17,377 proteins below 0.7 the corresponding numbers are 75 (0.43%) and 20 (0.12%). These differences are again highly significant (χ^2^ *P* = 2.4 × 10^−250^ and *P* = 2.4 × 10^−34^, respectively). Thus, while no single PASTA2 cutoff should be regarded as a gold-standard definition of amyloidogenicity, the strong enrichment of negative PASTA2 energies among proteins with higher AmyloGram scores provides useful orthogonal support for the practical utility of the 0.7 AmyloGram threshold.

**Figure 4:**
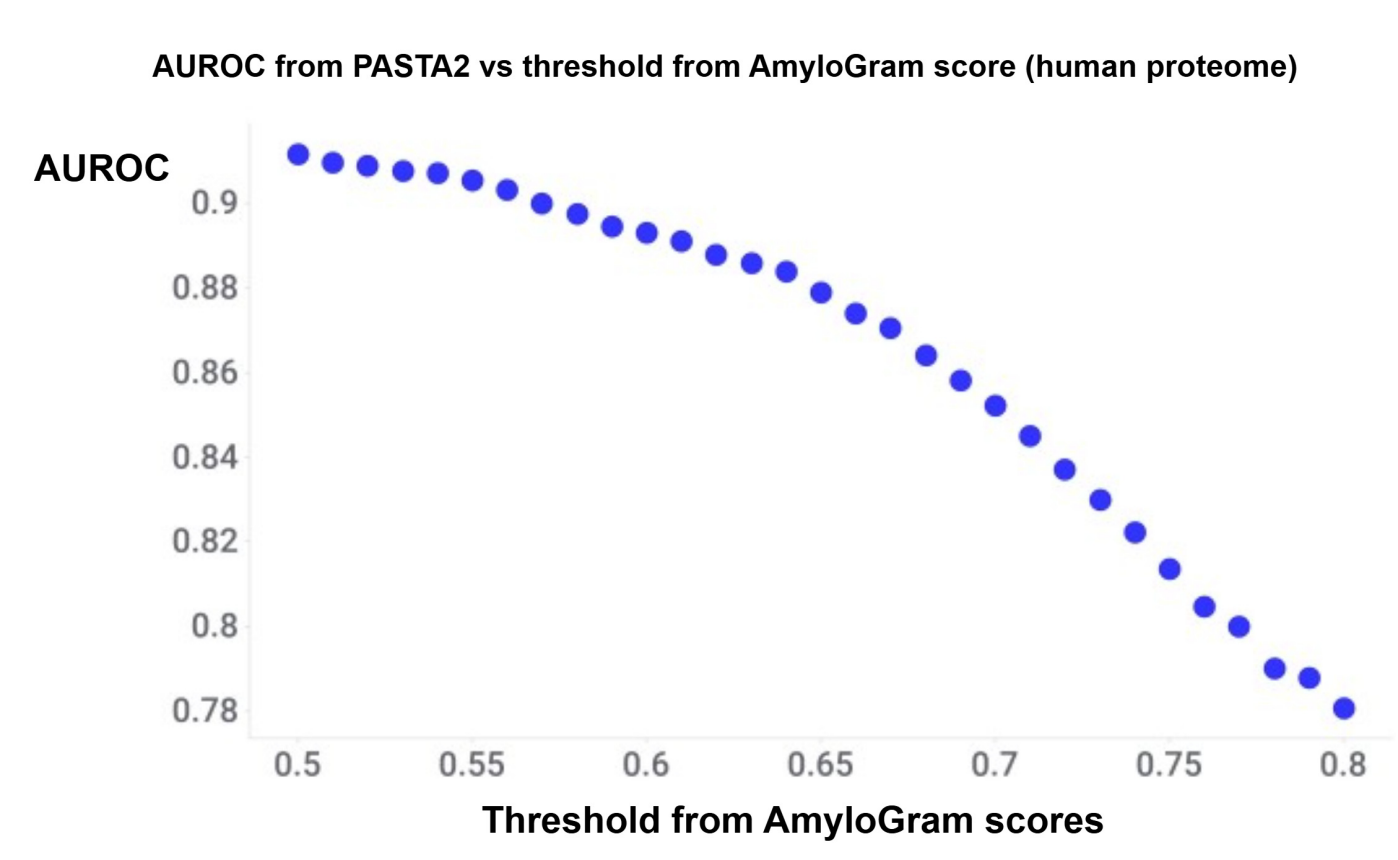
Relationship between the area under the reciever-operator curve (AUROC) values when PASTA2 average negative free energies are used at different values of the AmyloGram score threshold that is varied to admit a binary output Area-under-the-curve values obtained when PASTA2 average negative free energies are used descriptively to classify proteins after binarising AmyloGram at thresholds varied from 0.50 to 0.85. This is not a formal ROC analysis against experimental ground truth; rather, it is a comparison between two computational predictors applied to the same human-proteome dataset. The figure is therefore intended only to illustrate how the apparent discriminatory relationship between the two predictors changes as the AmyloGram threshold is altered. The broad maximum in the region of approximately 0.65–0.70 is consistent with the previously adopted operational threshold of 0.7, but should not be interpreted as demonstrating a unique optimal cutoff.

Of the 66,191 proteins with an AmyloGram score of 0.7 or greater, some 4208 have an average free energy lower than −10 PEU and 454 lower than −20 PEU, whereas for those with an AmyloGram score of 0.7 or lower the numbers are only 49 and 18, respectively. This again shows the striking differences in amyloidogenicity as calculated using PASTA2 between proteins based on whether their AmyloGram scores are greater or less than 0.7.

Another strategy we considered for relating PASTA2 free energies and AmyloGram scores was to use the AmyloGram score as a binary threshold and use the PASTA2 scores as inputs to a Receiver Operator Characteristic (ROC) curve. We varied the threshold for the AmyloGram score from 0.5 to 0.85, with the results shown in Figure 4. The fact that the PASTA2 average free energy can vary widely for a given AmyloGram score suggests that there is no specific threshold that might be seen as optimal based on this reasoning, though given the knee in the curve of Figure 4 around an AmyloGram score of ∼0.65, our earlier choice of 0.7 might then even be seen as conservative. We stress, however, that this is only a descriptive comparison between two predictors and not a formal ROC analysis against experimental ground truth. The broadness of the relationship between PASTA2 average free energy and AmyloGram score means that Figure 4 should be read only as showing that thresholds in the region of 0.65–0.7 behave sensibly, not as proving a unique optimal threshold.

We next downloaded the 125 proteins curated at AmyPro (https://amypro.net/#!/) [98] and assessed both their PASTA2 free energy values and their AmyloGram scores. Eight proteins in AmyPro were labelled as non-amyloid and were removed, leaving 117 as ‘amyloid’. The former small control set means that any inferential statistics should be interpreted with caution, but the data remain useful for checking whether known amyloids tend to occupy the higher end of the AmyloGram distribution. The median minimum and average PASTA2 free energies for the amyloid proteins were −7.11 and −4.14 PEU, whereas the corresponding values for the non-amyloids were −4.63 and −2.68 PEU. The median AmyloGram scores for the amyloid and non-amyloid proteins were 0.800 and 0.407, respectively. We also summarised their source-organism distribution and sequence lengths in the AmyPro supplementary table to make the composition of this validation set more transparent. Full details are given in Supplementary Table S1.

**Figure 5:**
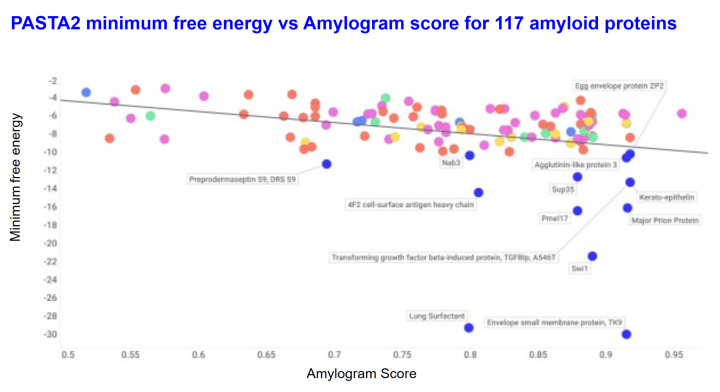
AmyPro curated amyloid proteins: AmyloGram scores versus PASTA2 minimum free energies Scatter plot for the 117 proteins curated in AmyPro as amyloid in nature, comparing AmyloGram score with PASTA2 minimum free energy. Each point represents one curated amyloid protein. A line of best fit is shown, but the relationship is weak (*r*^2^ = 0.10), indicating that even within a set of experimentally implicated amyloid proteins the two predictors do not agree closely on rank ordering. Proteins with PASTA2 minimum free energy ≤−10 PEU are labelled individually to highlight the subset predicted by PASTA2 to be especially aggregation-prone. This figure is included to show the range of scores occupied by curated amyloids, not to imply that one predictor can substitute for experimental annotation. The colours do not have a meaning save that those is dark blue are those labelled.

**Figure 6:**
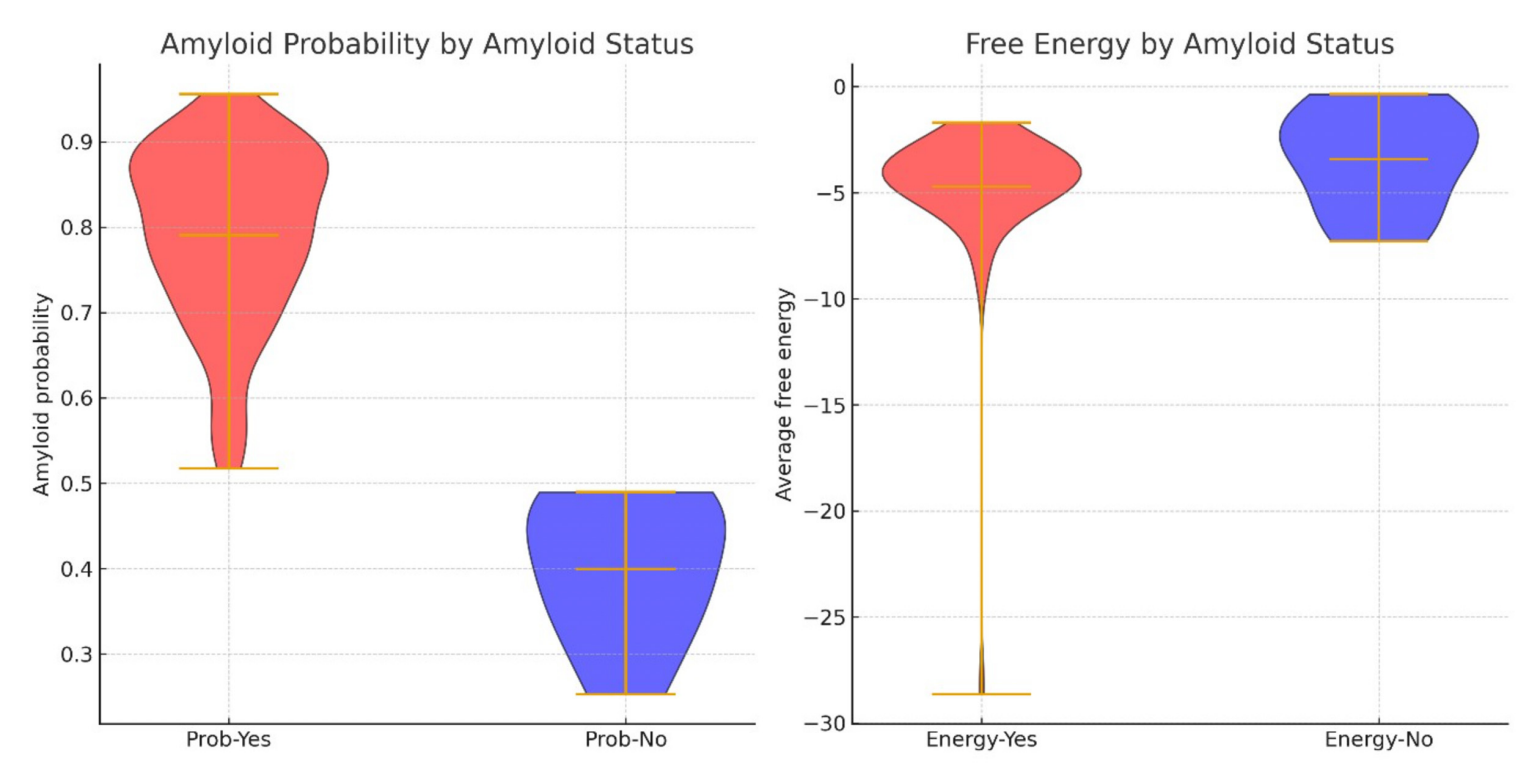
AmyPro amyloid-status comparison for AmyloGram and PASTA2 Comparison of predictor outputs against AmyPro curation status for the 125 proteins analysed (117 annotated as amyloid and 8 annotated as non-amyloid). ‘Amyloid status encoding’ refers to the binary AmyPro annotation (amyloid vs non-amyloid). For each protein, AmyloGram probability and PASTA2 average free energy are plotted against this status to show how the two predictors distribute across the curated positive and negative sets. Higher AmyloGram scores indicate greater predicted amyloidogenicity, whereas more negative PASTA2 free energies indicate greater predicted aggregation propensity. The figure is intended as a visual summary of the tendency for curated amyloids to occupy higher AmyloGram and more negative PASTA2 ranges than the non-amyloid controls, while also making clear that the non-amyloid comparator set is very small and heavily imbalanced. Yes and No reflect the amyloid status so Prob-Yes and Prob-No are the amyloid probabilities for the two classes amyloid and non-amyloid, while similarly Energy-yes and Energy-No are the average free energies for the two amyloid classes.

The comparison between PASTA2 minimum free energy and AmyloGram score is plotted in Figure 5, while Figure 6 shows the data for the amyloid and non-amyloid proteins as a violin plot. While this analysis shows a tendency of separation it should be considered with caution as there are only eight non-amyloid proteins in this dataset. On the other hand the separation between the two groups is much stronger when considering the AmyloGram scores.

### AmyloGram and AMYPred-FRL amyloidogenicity prediction comparison

**Figure 7:**
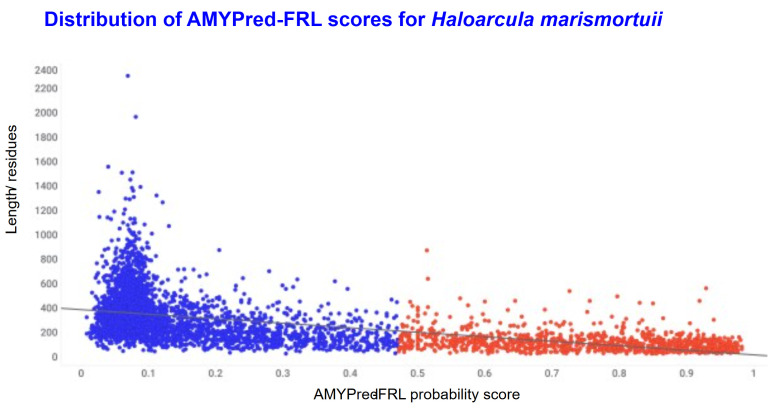
Distribution of AMYPred-FRL outputs in the archaeal proteome of *H. marismortui* Scatter plot showing AMYPred-FRL probability scores for the 4234 proteins of *H. marismortui* (strain ATCC 43049), shown as a function of protein length. Each point represents one protein. Proteins classified by the AMYPred-FRL server as amyloid at its default internal threshold are shown in red; those not classified as amyloid are shown in the other plotting colour. The fitted line has *r*^2^ = 0.28. The purpose of this figure is descriptive: it shows the internal behaviour of AMYPred-FRL on a representative archaeal proteome and makes explicit what is meant by the server’s probability output and binary amyloid/non-amyloid call.

**Figure 8:**
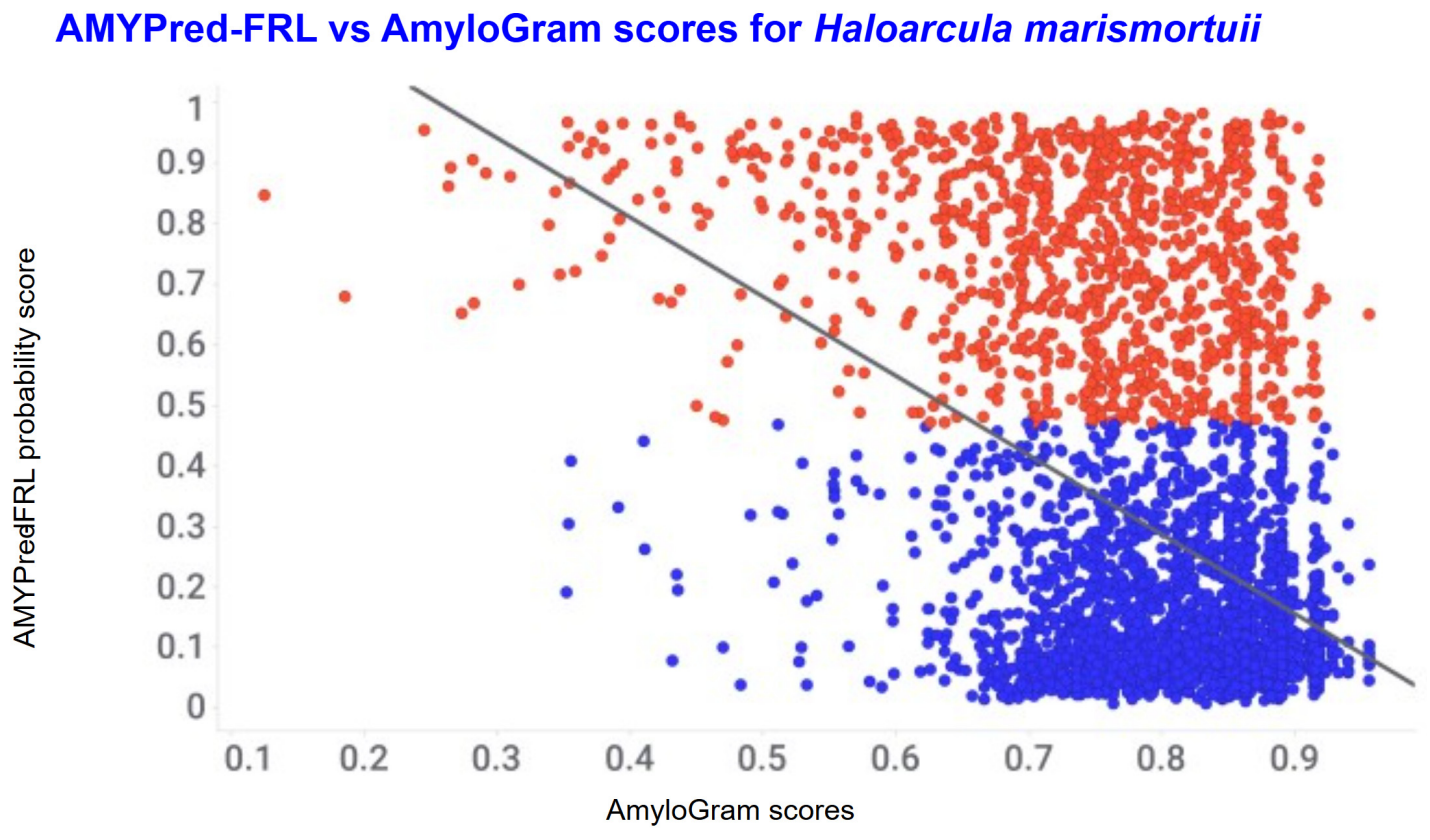
Comparison of AMYPred-FRL and AmyloGram in *H. marismortui* Scatter plot comparing AMYPred-FRL probability scores with AmyloGram scores for the same 4234 proteins from *H. marismortui* analysed in Figure 7. Each point represents one protein. Proteins classified as amyloid by the AMYPred-FRL server at its default threshold are highlighted in red. The fitted linear regression is weak and not positively sloped overall (*r*^2^ = 0.19), indicating only limited concordance between the two predictors in this proteome.

An alternative to PASTA2 is provided by AMYPred-FRL [99] (https://pmlabstack.pythonanywhere.com/AMYPred-FRL), whose output is a ‘probability score’ for being an amyloid. As an example of an archaeon, that happened to be the first in our list, with 4234 proteins (running 20x more quickly than an analysis of the human proteome), we ran the *Haloarcula marismortui* (strain ATCC 43049) proteome through AMYPred-FRL. Here, the purpose was not to establish a new threshold, but to ask whether a third predictor with a rather different training strategy gave outputs broadly concordant with AmyloGram. The results are shown in Figures 7 and 8.

Clearly the likelihood of being labelled an amyloid by AMYPred-FRL is determined by the internal threshold set by the AMYPred-FRL program, that set by default (and illustrated) being 0.47. However, even with this comparatively low setting only 1055 out of the 4234 proteins are labelled as amyloid, making it highly unlikely that the AMYPred-FRL score would correlate well with the AmyloGram scores. To this end, Figure 8 shows the lack of a positive relationship between the AMYPred-FRL probabilities for this proteome and the AmyloGram score for the same proteome; indeed the (relatively weak) correlation has a negative slope. Consequently, the AMYPred-FRL outputs are not directly comparable to AmyloGram in a simple one-to-one sense. Figure 8 confirms that the relationship between the AMYPred-FRL probabilities and the AmyloGram scores in this proteome is weak and not positively sloped overall (*r*^2^ = 0.19 for the fitted line). We therefore treat AMYPred-FRL here mainly as a reminder that different predictors need not agree strongly, not as a basis for any strong biological conclusion. This figure is included primarily to show that different amyloid-prediction methods do not need agree strongly, and that the AMYPred-FRL comparison is a contextual benchmarking exercise rather than a basis for a major biological conclusion.

### Amyloidogenicity of the proteomes of various taxa

**Figure 9:**
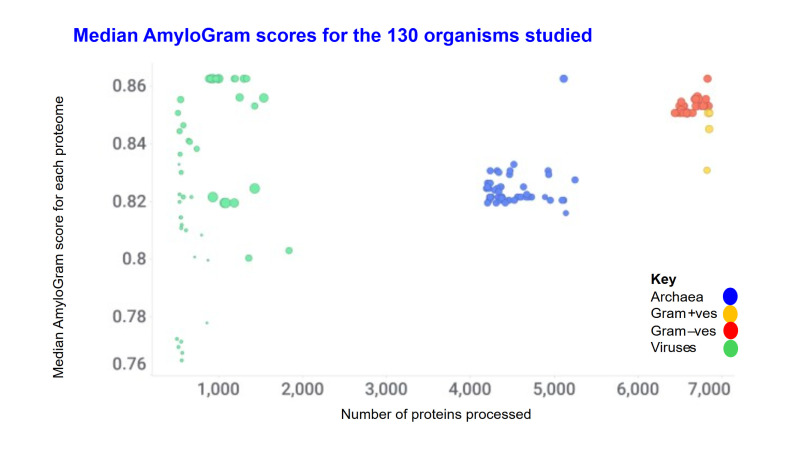
Proteome-wide AmyloGram medians across 130 organisms Bubble plot summarising organism-level median AmyloGram scores for 130 proteomes comprising 49 archaea, 31 bacteria, and 50 viruses (475,999 proteins in total). Each point represents one organism. The *x*-axis gives the number of proteins analysed for that proteome, the *y*-axis gives the median AmyloGram score for all proteins in that proteome, point colour denotes taxonomic domain (archaea, Gram-negative bacteria, Gram-positive bacteria, or viruses), and point size encodes average protein length. All underlying organism-level values are listed in Supplementary Table S1. The figure shows that high median predicted amyloidogenicity is widespread across all four taxonomic groupings, with modest but statistically significant differences between them (Kruskal–Wallis *P* = 5.0 × 10^−8^). This is the central evolutionary figure of the paper.

We next downloaded FASTA files for the proteomes of a series of organisms (listed in Supplementary Table S2), and assessed (the distributions of) their proteome properties and AmyloGram scores. Because of limitations on the availability of computational resources, we confined ourselves to 50 viruses, 49 archaea, and 31 bacteria, but we are confident in the statistical conclusion as we analysed no fewer than 475,999 proteins in all. A numerical summary is given in Table 1 and a visual illustration in Figure 9. These analyses address the second motivating question of the study, namely whether high predicted amyloidogenicity is confined to the human proteome or is also observed across evolutionarily more ancient taxa.

**Table 1:** Summary statistics for selected properties of proteomes of the 130 organisms studied

Taxonomic domain	Average protein count (integer, ±SD)	Average protein length (residues, integer ± SD)	Average AmyloGram score (±SD)	Median AmyloGram score
Archaea	4510 ± 295	288 ± 9	0.802 ± 0.006	0.822
Gram-negative bacteria	6639 ± 136	367 ± 7	0.832 ± 0.003	0.853
Gram-positive bacteria	6838 ± 12	312 ± 11	0.819 ± 0.01	0.851
Viruses	848 ± 12	205 ± 11	0.805 ± 0.027	0.825
All 130 organisms	3662 ± 2368	301 ± 60	0.810 ± 0.021	0.828

From the above, we conclude that even proteins from early evolutionary taxa are on average highly amyloidogenic. At the organism level, the median AmyloGram scores differed significantly across the four taxonomic groupings (Kruskal–Wallis *P* = 5.0 × 10^−8^), although the absolute differences between some clades were modest. In particular, Gram-negative bacteria tended to have slightly higher organism-level medians than archaea, Gram-positive bacteria and viruses, whereas archaea and viruses overlapped more extensively. Thus, the data indicate broad conservation of high predicted amyloidogenicity, while not requiring that all domains be sharply separated from one another.

### Analysis of random sequences

**Figure 10:**
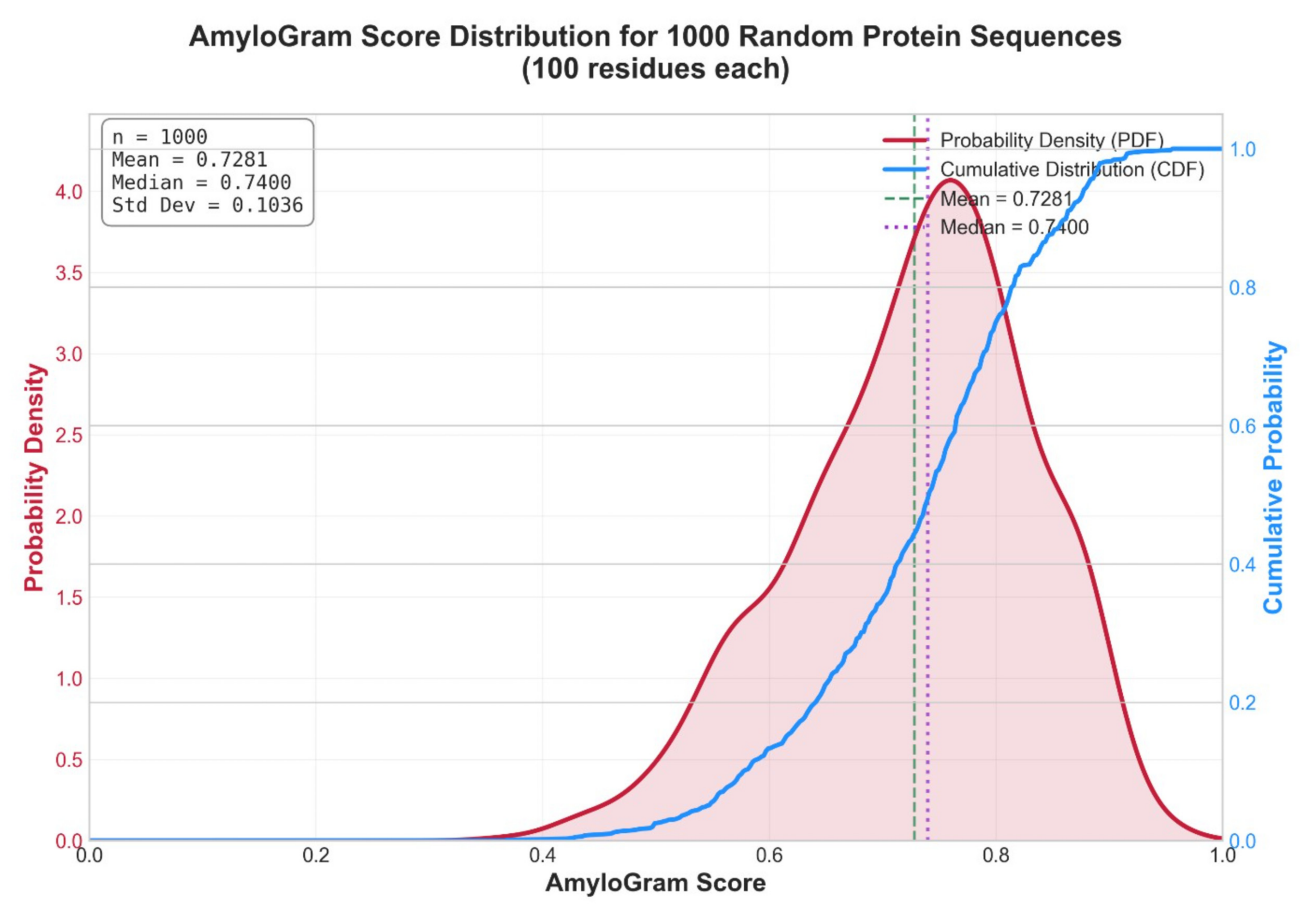
AmyloGram score distribution for random 100-residue sequences Distribution of AmyloGram scores for 1000 independently generated random protein-like sequences, each 100 residues long. At each position, one of the 20 standard amino acids was sampled uniformly, with no additional compositional or structural constraints. The figure is included as a simple unevolved control against which the evolved proteomes in Figure 9 can be compared. Although some random sequences achieve very high AmyloGram scores, the overall distribution is shifted to lower values than the proteome medians from natural organisms. For these random sequences, the mean score was 0.728, the median was 0.740, and the standard deviation was 0.104.

While the organism-level median AmyloGram score for each of these taxonomic domains was greater than 0.8, the question then arises as to how different these distributions would be if we simply ran unevolved random sequences through AmyloGram. For speed, we used 1000 examples of 100mers, with the results shown in Figure 10. The sequences are given in Supplementary Table S3.

**Figure 11:**
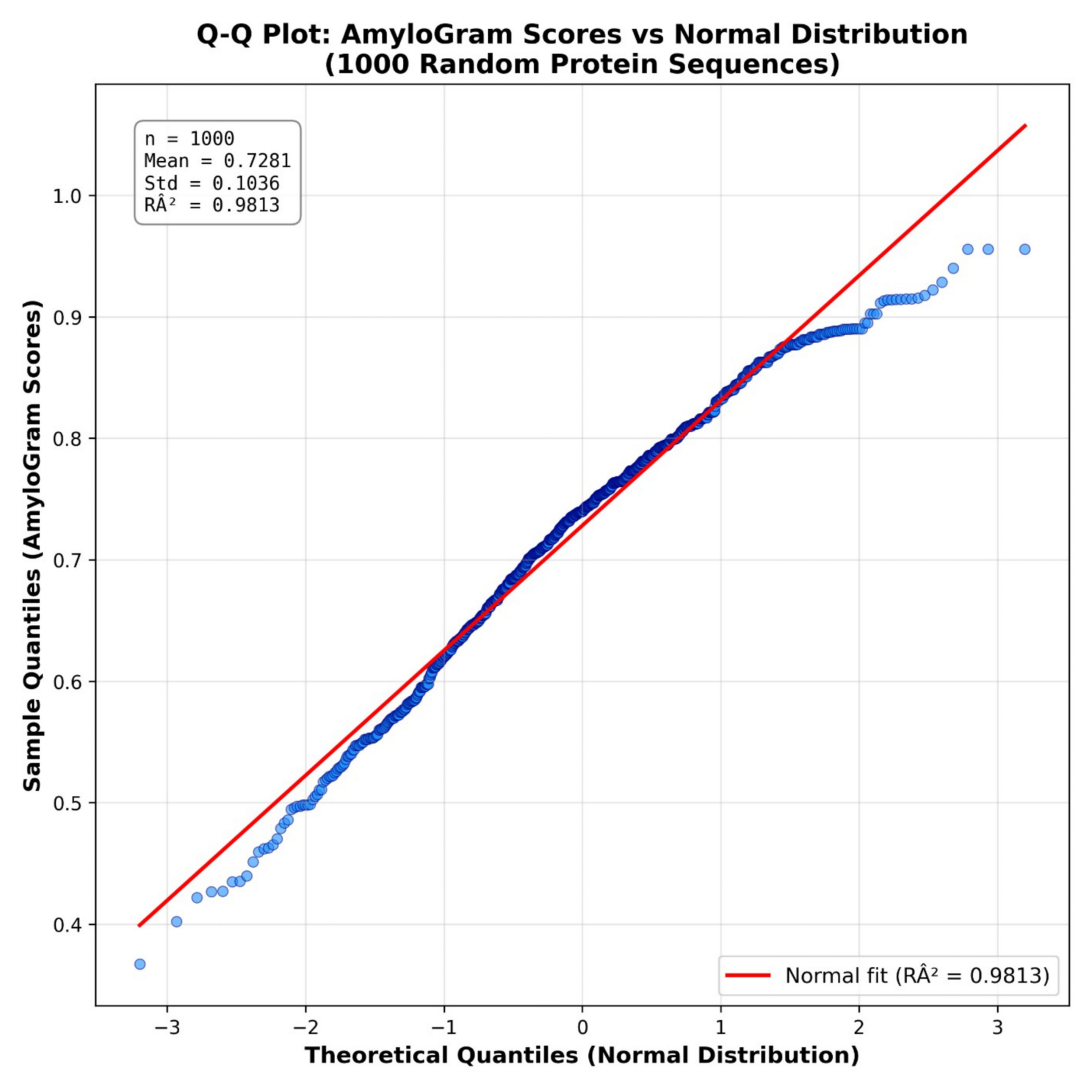
QQ plot for AmyloGram scores of random sequences Quantile–quantile plot for the 1000 random-sequence AmyloGram scores shown in Figure 10, comparing their observed distribution with that expected under normality.

The random sequences had a mean score of 0.728, a median of 0.740 and a standard deviation of 0.104. Comparison of the random-sequence scores with the organism-level proteome medians gave a highly significant difference (Mann–Whitney U *P* = 3.45 × 10^−36^). The QQ plot in Figure 11 shows that the random-sequence distribution is itself non-normal, with an extended upper tail. This might have been taken to mean that evolved proteins are enriched for high-scoring sequences relative to this simple random control. Whether this reflects ancient inheritance, general biophysical constraints associated with folding, or some degree of model bias cannot be resolved from the present data alone. In practical terms, this means that simple random sequences can occasionally receive very high AmyloGram scores, but that these scores are unevenly distributed rather than forming a symmetric Gaussian background. This behaviour supports the cautious interpretation adopted in the text: evolved proteomes are enriched for high-scoring sequences relative to this simple random control, but the comparison does not by itself establish a unique evolutionary explanation.

### Using sequences reflecting known amino acid distributions

We note that the distributions of amino acid residues in proteins are not identical, of course, and also differ between different organisms [100]. We ran the same unevolved sequences but now using the human proteome distributions given in the Supplementary Information to [100]. These 100mer sequences had a mean score of 0.729, a median of 0.741, and a standard deviation of 0.11, numbers almost identical to those of the random sequences, consistent with a view that those amino acids with greater or lesser amyloidogenic tendency than the average effectively balance out.

**Figure 12:**
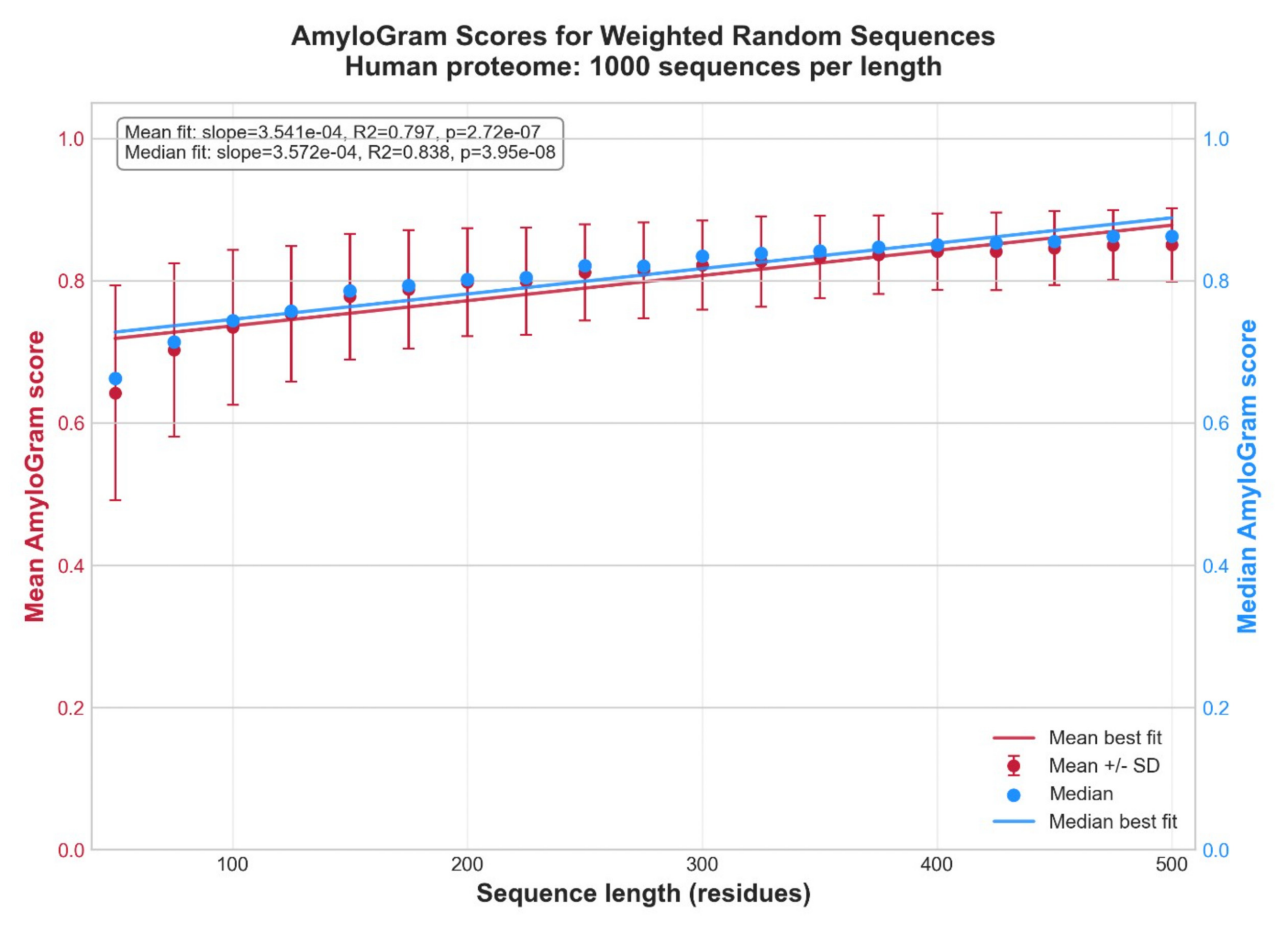
Relationship between the mean ± SD (red) and median (blue) AmyloGram scores for proteins with sequences that were random but weighted according to the amino acid frequencies in the human proteome [100], and the sequence length used Each length was generated 1000 times and run through AmyloGram. The Amylogram scores typically exceed 0.8 for lengths over 250 residues.

**Figure 13:**
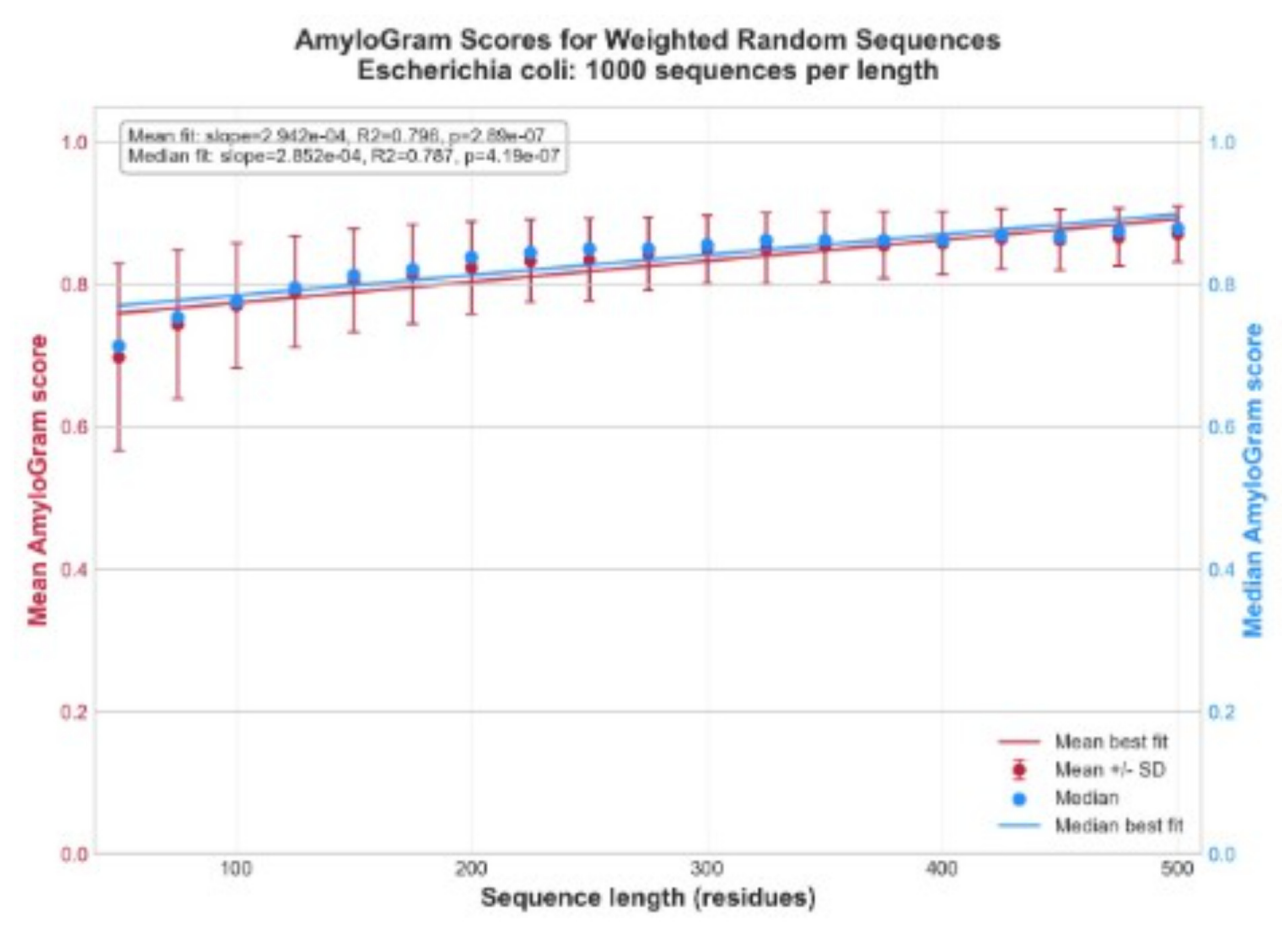
Relationship between the mean ± SD (red) and median (blue) AmyloGram scores for proteins with sequences that were random but weighted according to the amino acid frequencies in the *E. coli* proteome [100], and the sequence length used Each length was generated 1000 times and run through AmyloGram. The Amylogram scores typically exceed 0.8 for lengths over 175 residues.

Although there is only a relatively weak dependency of AmyloGram score on sequence length (Figure 10 of [35] and Figure 4 of [48]), it is not negligible, and a referee pointed out that a better comparison would take sequence length into account. The protein compositions most different from each other in reference [100] are those of humans and *Escherichia coli*. The median residue length in the human proteome is ca 375 and that for *E. coli* is ca 300. We ran the weighted sequence distributions for both human and *E. coli* distributions as given in the Supplementary Information to [100], varying the length of the ‘proteins’ from 50 to 500 residues (each again run 1000 times). Under these circumstances, the mean (±SD) and median of 375mers were 0.837 ± 0.055 and 0.847 for the human proteome and mean 0.855 ± 0.047 and median 0.863 for *E. coli*. This implies a relative independence for evolutionary position relative to the non-negligible influence of protein residue length. The length dependence for the two proteomes is given in Figures 12 and 13, respectively.

Overall, we can conclude that the sequences selected for during natural evolution, under circumstances that are clearly adaptive, also tend to favour amyloidogenesis, whether as a kind of ‘passenger’ effect or because (perhaps more likely) the biophysical features they possess (such as hydrophobicity [101–103] and β-sheet-forming tendency [104–106]) are in at least some cases relevant both to the formation of secondary structures and to amyloidogenicity.

## Discussion and conclusions

Our previous analyses [48] used proteins annotated in UniProt as known amyloids to indicate that any protein with an AmyloGram score exceeding 0.7 was likely to be amyloidogenic (and in fact some known amyloidogenic proteins had AmyloGram scores below this [48]). On that basis, 79.2% of the human proteome was seen as amyloidogenic, and the median AmyloGram score for that proteome was 0.813 [48]. We recognise that β-sheet-forming and hydrophobic sequences tend to be more amyloidogenic (something that follows from the β-sheet-forming propensity of individual amino acids), but that the Amylogram score scales only weakly with protein length (Figure 10 of [35] and Figure 4 of [48]). Specifically, ‘the principal sequence determinants used by AmyloGram to predict amyloidogenicity are hydrophobicity, β-sheet propensity, and reduced amino acid flexibility’ [35].

The first purpose of the present work was therefore to determine how far this simple AmyloGram score is supported by orthogonal prediction frameworks. The PASTA2 comparisons show that proteins above the AmyloGram threshold are far more likely also to have strongly negative PASTA2 free energies, and the AmyPro data show that curated amyloid proteins occupy markedly higher AmyloGram values than the small curated non-amyloid set. At the same time, the weak relationship with AMYPred-FRL underlines that no single predictor should be treated as an unquestioned gold standard.

Ancient proteins in an evolutionary sequence are well known to have a tendency to be more stable to heat and to other stresses, something that is generally assumed to relate to geologically early conditions [107–123]. On the basis of such differences, they might in principle be differentially amyloidogenic, and there is evidence both for [124,125] and against [126] a positive and mechanistic linkage between thermostability and amyloidogenicity in different examples. Thus, the second purpose of the present work was evolutionary. To this end, we ran the proteomes of 130 organisms (viruses, archaea, Gram-negative and -positive bacteria) through AmyloGram, representing a total of 475,999 proteins. The median AmyloGram scores for the different clades ranged from 0.822 to 0.853, exceeding that of the human proteome (0.813). These values are therefore consistent with the idea that high amyloidogenic potential is a general property of evolved proteins rather than an idiosyncrasy of the human proteome. However, several explanations remain possible.

- The pattern may indeed reflect an ancient origin of widespread amyloidogenic potential;
- it may reflect parallel evolution under common physicochemical constraints,
- it may reflect bias in a predictor trained largely on contemporary proteins, or
- in our view most likely the pattern may reflect the simple biophysical tendency of certain residues and sequences to induce amyloidogenicity.

Consequently, we regard the present findings as consistent with, rather than proving, an early evolutionary origin of amyloidogenic potential.

More specifically, the high scores observed here may arise because the physicochemical features that contribute to ordinary protein structure and function, such as hydrophobicity and β-sheet-forming propensity, also contribute to amyloidogenicity. In that sense, amyloidogenic potential may be an unavoidable fellow-traveller of folded protein chemistry rather than a trait selected specifically for amyloid formation. Addressing that possibility properly will require additional analyses of sequence composition, secondary-structure propensity, protein length and matched non-amyloid controls, which lie beyond the scope of the present paper.

Overall, our data support three general conclusions. First, AmyloGram scores are positively related to those from PASTA2, and proteins with higher AmyloGram scores are strongly enriched for more negative PASTA2 free energies. Secondly, curated amyloid proteins in AmyPro tend to have higher AmyloGram scores than do those in the small curated non-amyloid set, although the severe class imbalance demands caution. Third, high predicted amyloidogenicity is widespread across the proteomes analysed here. These findings are therefore consistent with a very early and widespread evolutionary emergence of amyloidogenic potential, while stopping short of proving that such potential must have a single ancient origin. Overall, we consider that our findings here provides very strong support for the statement that ‘most natural proteins are potentially amyloidogenic’. This can be seen as underpinning the extensive amyloidogenic cross-seeding (reviewed in [30–32,34,35,127]) that can be observed during amyloid formation in complex biological milieux.

## Methods

### Publicly available datasets

We downloaded a variety of proteomes from the relevant parts of UniProt, using either organism names or their ‘taxonomy ID’ (for example, *E. coli* K-12 strain MG1655,with the taxonomy ID 511145 is at https://www.uniprot.org/taxonomy/511145). We also used AmyPro (https://amypro.net/#!/), a database of validated amyloid precursor proteins and their amyloidogenic sequence regions, was used for validation [98]. For the AmyPro set analysed here there were 117 proteins curated as amyloid and 8 curated as non-amyloid. We summarised these proteins additionally in terms of their source organisms and sequence lengths, to make clear both the utility and the limitations of this heavily imbalanced validation set. Because the AmyPro non-amyloid set contains only eight proteins, results involving that comparison are interpreted cautiously.

### Amyloidogenicity prediction

AmyloGram was run by pasting the relevant FASTA sequences into its website http://biongram.biotech.uni.wroc.pl/AmyloGram/ for protein numbers below 50. For larger numbers, the CRAN R-package AmyloGram (version 1.1) https://cran.r-project.org/web/packages/AmyloGram/index.html was called either from RStudio (R 4.4.1) directly or from a Python 3.12 script within the Cursor environment. Other online tools for amyloidogenicity prediction included the PASTA 2.0 server (http://protein.bio.unipd.it/pasta2/) [83] and AMYPred-FRL [99] (https://pmlabstack.pythonanywhere.com/AMYPred-FRL). These were used as external comparators; their methodological descriptions are therefore given here rather than in the Results. PASTA2 estimates the stability of putative cross-β pairings and returns energy values in PEU, with more negative values indicating greater predicted aggregation propensity. AMYPred-FRL returns a probability score and, at its default setting, an internal binary classification. Statistical analyses were carried out in Python using chi-square or Fisher exact tests for contingency tables, Mann–Whitney U tests for two-group comparisons, and Kruskal–Wallis tests for comparisons across taxonomic domains.

### Random sequence generation

For the random-sequence analysis, we generated 1000 independent sequences by sampling each position uniformly from the 20 standard amino acids; no additional compositional or structural constraints were initially imposed. These sequences are provided as supplementary material. We also include sequences biased to display the known residue frequencies for the relevant organism. Analysis of the distributions of AmyloGram scores was performed in Excel or using Python, with some of the visualisations using the program Spotfire.

## Supplementary Material

Supplementary Tables S1-S3

## Data Availability

Data are available from the sources cited and in Supplementary Tables S1–S3.

## Open Access

Open access for this article was enabled by the participation of the University of Liverpool in an all-inclusive Read & Publish agreement with Portland Press and the Biochemical Society under an agreement with JISC.

## Abbreviations

PEU: PASTA Energy Unit
ROC: Receiver Operator Characteristic
